# Association of Maternal Age to Development and Progression of Retinopathy of Prematurity in Infants of Gestational Age under 33 Weeks

**DOI:** 10.1155/2014/187929

**Published:** 2014-04-30

**Authors:** Atsuro Uchida, Masayuki Miwa, Hajime Shinoda, Takashi Koto, Norihiro Nagai, Hiroshi Mochimaru, Yohei Tomita, Mariko Sasaki, Kazushige Ikeda, Kazuo Tsubota, Yoko Ozawa

**Affiliations:** ^1^Department of Ophthalmology, Keio University School of Medicine, 35 Shinanomachi, Shinjuku-ku, Tokyo 160-8582, Japan; ^2^Department of Pediatrics, Keio University School of Medicine, 35 Shinanomachi, Shinjuku-ku, Tokyo 160-8582, Japan

## Abstract

*Aim*. To find predictive and indicative markers of risk for development of retinopathy of prematurity (ROP) and its progression to the stage requiring laser treatment, in premature infants whose gestational age (GA) was under 33 weeks. *Methods*. We retrospectively reviewed medical records of 197 premature infants born in 2005–2010 whose GA < 33 weeks and underwent eye screening at Keio University Hospital. The association between candidate risk factors and development or progression of ROP was assessed. *Results*. Among the 182 eligible infants (median GA, 29.1 weeks; median birth weight (BW), 1028 g), 84 (46%) developed any stage of ROP, of which 45 (25%) required laser treatment. Multivariate analysis using a stepwise method showed that GA (*P* = 0.002; 95% confidence interval (CI), 0.508–0.858), BW (*P* < 0.001; 95% CI, 0.994–0.998), and lower maternal age (*P* = 0.032; 95% CI, 0.819–0.991) were the risk factors for ROP development and GA (*P* < 0.001; 95% CI, 0.387–0.609) and lower maternal age (*P* = 0.012; 95% CI, 0.795–0.973) were for laser treatment. The odds ratio of requiring laser treatment was 3.3 when the maternal age was <33 years. *Conclusion*. ROP was more likely to be developed and progressed in infants born from younger mother and low GA.

## 1. Introduction

Recent progress in biotechnology and medical care has increased the number of surviving preterm newborns worldwide [1, 2]. Advances in perinatal medical care have increased the survival rate of infants delivered prematurely; more than 80% of very low birth weight (VLBW) infants survive in developed countries [3, 4]. These preterm infants, however, are a vulnerable population, at high risk for developmental disorders in multiple organs. Retinopathy of prematurity (ROP) is a frequent disorder in these survivors.

ROP has been associated with excessive oxygen use shortly after birth. ROP develops in two phases [5–8]. Phase I is a vasoobliteration phase, resulting from the relatively hyperoxic environment induced by oxygen supplementation therapy. This condition suppresses vascular endothelial growth factor (VEGF), thereby inhibiting normal retinal growth and promoting capillary obliteration. During phase II, the cessation of oxygen supplementation therapy causes relatively hypoxic conditions in the retina, which dramatically induces VEGF, resulting in abnormal neovascularization. Today, oxygen supplementation is monitored to avoid excessive administration; however, ROP continues to be an important cause of preventable blindness. It is estimated that over 50,000 children per year worldwide still become blind or are visually impaired due to ROP [1].

Appropriately timed laser treatment is the main therapeutic approach for preventing blindness in these infants, although clinical trials also support the use of anti-VEGF drugs to regress ROP [9]. With any method, the treatment should be performed at the most appropriate disease stage. To improve their prognosis, we sought to identify infants who would require laser treatment and to apply the treatment at the optimal time. Because the number of preterm infants is growing and those who need to be screened for their ROP are increasing, particularly in the center hospitals, and the fundus examination may increase the stress of infants in incubators, it would be helpful to both the doctors and the infants to find predictive and indicative factors for severe ROP requiring laser treatment. Such factors will help us determine which infants need to be followed closely for severe ROP who requires laser treatment and minimize unnecessary examinations for infants who are not at high risk.

Previous reports have documented the major risk factors for ROP [5, 10], which include extremely low gestational age (GA), low birth weight (BW), and excessive oxygen supplementation. In this study, we retrospectively reviewed 182 preterm (GA < 33 weeks) infants who were routinely asked to perform eye screening from Department of Pediatrics in our hospital as high risk infants, to elucidate risk factors for ROP development and also its progression.

## 2. Materials and Methods

### 2.1. Study Population

The study followed the tenets of the Declaration of Helsinki and was approved by the ethics committee of the Keio University School of Medicine. The study included infants hospitalized in the neonatal intensive care unit in Keio University Hospital, the tertiary neonatal hospital in Japan, with a GA < 33 weeks, and undergone ophthalmological examinations at the neonatal intensive care unit by the retina specialists of the Retina Division Clinic of Department of Ophthalmology, in Keio University Hospital between January 1, 2005 and December 31, 2010. The newborn infants were routinely screened for ROP by request from pediatricians. GA was determined by early ultrasound examination in the first trimester. Among 197 VLBW infants, 9 (5%) who died in their first weeks of life, 4 (2%) who had congenital anomalies, and 2 (1%) with insufficient medical information were excluded. Thus, 182 infants were analyzed, retrospectively.

### 2.2. Eye Examination and Indications for Laser Treatment

The pupils were dilated with topical 2.5% phenylephrine and 0.5% tropicamide. Ophthalmoscopy using a binocular indirect ophthalmoscope was performed using a lid speculum, a scleral indenter, and a 28-diopter lens (Volk, Mentor, OH). The initial examination was performed at 29 weeks of gestation or 3 weeks of chronological age, whichever was earlier. All infants were examined by either of 8 retina specialists (AU, HS, TK, NN, HM, YT, MS, and YO). Retinopathy was graded according to the international classification of ROP [11]. The maximum severity of ROP in either eye for an individual infant was recorded. The zone of vascularization (from I to III), presence or absence of plus or preplus disease, and the stage of ROP (stages 1–5) were evaluated. The examination was repeated every 1-2 weeks until the vascularization reached zone III, without any remaining zone I or zone II disease, or until the ROP had regressed. Laser treatment was performed when the eye had plus disease with any stage of ROP in zone I or with stage 2 or 3 ROP in zone II, or without plus disease but with stage 3 ROP in zone I, according to the Type 1 ROP criteria developed after the Early Treatment of ROP (ETROP) trial [12].

### 2.3. Data Collection

Data for the GA, BW, maternal age, small for GA (SGA) or appropriate for GA (AGA) infants, Apgar score (at 1 or 5 minutes), number of multiple birth, gender, and pulmonary surfactant supplementation were collected by reviewing the medical records. The neonates were classified as SGA or AGA according to their BW percentiles, defined by the neonatal growth standards of Ogawa et al. [13]. SGA infants were defined as those whose BW for GA was below the 10th percentile, and AGA infants were defined as those whose birth weight for GA was between the 10th and 90th percentiles (inclusive). In this study, no infants were large for gestational age whose BW or height was greater than 90th percentile.

For the purpose of data analysis, the infants were divided into those with no ROP and those with any stage of ROP. Also, the infants were divided into those with untreated ROP (no ROP or spontaneously regressed ROP) and those with severe ROP requiring laser treatment.

### 2.4. Statistical Analysis

Statistical analysis was performed using SPSS 17.0 (SPSS, Chicago, IL). Each group was compared with respect to continuous variables (GA, BW, maternal age, and Apgar score) and categorical variables (SGA/AGA, multiple birth, gender, and pulmonary surfactant). Continuous variables were first evaluated with the Shapiro-Wilk test and Kolmogorov-Smirnov test to confirm a normal distribution of the data. As the BWs, GAs, maternal age, and Apgar score did not follow a normal distribution, the Mann-Whitney *U* test was used to compare the baseline characteristics of each group. Categorical variables were compared using the chi-squared test. Subsequently, a multivariate logistic regression analysis by the stepwise method was performed to evaluate the independent risk factors of ROP. Statistical significance was determined at *P* < 0.05.

## 3. Results

Among 182 infants born preterm (<33 weeks of gestation), 84 (46%) developed any stage of ROP, of which 45 (25%) required laser treatment. The median GA and BW for the total cases were 29.1 weeks (range, 22.9 to 32.9) and 1028 g (range, 265 to 2,105), respectively.  Table 1 presents the univariate analyses of risk factors for ROP development. When compared to those without ROP, infants with any stage of ROP were of significantly lower GA and BW. Maternal age and Apgar score were significantly lower, and SGA and the usage of pulmonary surfactant were significantly more common among infants with any stage of ROP.  Table 2 shows the multivariate analysis of risk factors for ROP development using a stepwise method. In this analysis, after controlling for various potential confounders, significant variables were GA (*P* = 0.002), BW (*P* < 0.001), and maternal age (*P* = 0.032). Nonsignificant variables were SGA, Apgar score (1 and 5 minutes), multiple birth, gender, and the usage of pulmonary surfactant.

Table 3 shows the univariate analyses of risk factors for laser treatment. Significant variables were in common with the risks of ROP development except SGA. We found maternal age was again included in the risk. We compared the incidence of laser treatment between infants with GA less than the 25th percentile whose mothers' age were lower than the 25th percentile and higher than the 75th percentile. There was a trend of higher risk in the infants with GA less than the 25th percentile from younger mothers (70%) than the older mothers (56%), although there was no statistical significance (*P* = 0.43, data not shown).  Table 4 shows the results of multivariate regression analysis. By stepwise regression analysis, 2 variables were identified as significant risk factors for the development of severe ROP that lead to laser treatment; those were estimated GA (*P* < 0.001) and maternal age (*P* = 0.012). All the other variables were not significant. The odds ratio of requiring laser treatment was 3.3 when the maternal age was <33 years (data not shown in the table).

## 4. Discussion

We retrospectively analyzed incidence of development and progression of ROP in the GA < 33 weeks premature infants, who were hospitalized in the neonatal intensive care unit. In this study, we demonstrated that maternal age was inversely associated with the incidence of development and progression of ROP. Moreover, multivariate analysis indicated that this association was maintained after adjustment of known risk factors, such as gestational age and birth weight.

The overall risk for requiring laser treatment in our study was 25%, which was higher than retrospective study treated under the ETROP criteria in the United States (11%, BW < 1250 g, 2000–2005) [14] and large multicenter studies reported from Japan (10.8%, BW < 1500 g, 1984-1985) [15]. But the risk for requiring laser treatment in extremely low birth weight less than 1000 g in our study was 44.9% (data not shown), which was equivalent to a multicenter study held in Tokyo (41.0%, <1000 g, 2002) [16]. Thus, the criteria for the laser treatment would be appropriately followed, but the infants in this study might be more immature and, therefore, had higher risk for ROP progression, basically. This would be due to the nature of the neonatal intensive care unit in our hospital; it is a regional perinatal medical center and a tertiary referral hospital in Tokyo, where low-GA and/or very low-BW infants are routinely sent in order for the highly-specialized care of the neonates. Alternatively, there may be increasing number of immature infants who may not have survived until treatment, previously, but are rescued by high technology, in these days.

Advanced maternal age has become common in high income countries [17]. Woman's pursuit of higher education and carrier advancement and delayed marriage, as well as effective birth control and advances in assisted reproductive technology (ART), all contribute to the trend.

Maternal age has conventionally been seen as an important determinant of birth outcome [18]. It is well established that advancing maternal age is associated with subfertility, stillbirth, chromosomal abnormalities, multiple gestation, and abortion [19–21]. However, interestingly, maternal age was inversely associated with the incidence and progression of ROP in this study, suggesting the possibility that many of the infants with high maternal age might not have survived, and infants who overwhelmed the risk of pregnancy failure in spite of the high maternal age and were born alive might have less biological problems and, therefore, succeeded in vessel development in the retina, avoiding ROP. In contrast, younger mothers having premature infants would have had critical events which disrupted the pregnancy, and the event may have affected the infant's development, increasing the incidence of ROP.

There is a report that infants born with advanced maternal age may be more likely to develop ROP [22], inconsistent with our results, while others failed to find any association between maternal age and ROP [23, 24]; Wu and colleagues [22] conducted GA-matched and sex-matched case-control study and reported an increased risk of ROP in infants whose maternal age is older among the infants born before 37 weeks of gestation. The reason for this discrepancy is currently not known, but the difference of their study from ours is that while our study included all the infants routinely examined, their study was performed in the selected infants matching GA and sex and, therefore, included more infants whose GA and BW are larger.

Although SGA and the usage of pulmonary surfactant have been previously reported to be an independent risk factor associated with ROP [25–27], it was not a significant factor in the present study.

In conclusion, our findings suggest that, among preterm infants born GA <33 weeks, low GA and decreased maternal age were independent risk factors for severe ROP requiring laser treatment, not only for ROP development. Because these risk factors can be identified at birth, infants requiring circumspect management, such as frequent examinations, may be predicted at birth. Our findings provide important clinical insight for improving the management of ROP, although further studies are required.

## Figures and Tables

**Table 1:** Univariate analyses of risk factors for developing any stage of ROP in gestational age <33 weeks infants.

Variable	Without ROP (*n* = 98)	Any stage of ROP (*n* = 84)	*P* value	Odds ratio	95% CI
Gestational age (weeks)^†^	31.1 (29.3–32.0)	26.7 (25.5–28.3)	<0.001*	0.474	0.386–0.584
Birth weight (g)^†^	1356 (1057–1635)	761 (596–919)	<0.001*	0.995	0.993–0.996
Maternal age (years)^†^	33 (30–37)	31 (28–35)	0.009*	0.924	0.869–0.984
SGA (*n*, %)	26 (27)	36 (43)	0.021*	2.077	1.114–3.872
Apgar score; 1 min^†^	6 (4–8)	3 (1–6)	<0.001*	0.735	0.647–0.834
Apgar score; 5 min^†^	8 (6–9)	6 (4–8)	<0.001*	0.651	0.548–0.774
Multiple birth (*n*, %)	28 (29)	20 (24)	0.467	0.781	0.401–1.521
Gender; Male (*n*, %)	56 (57)	39 (46)	0.149	0.650	0.362–1.168
Pulmonary surfactant (*n*, %)	32 (33)	54 (64)	<220.001*	3.713	2.008–6.862

CI: confidence interval; ROP: retinopathy of prematurity; SGA: small for gestational age.

*P < 0.05.

^†^Median (interquartile range).

**Table 2:** Multivariate logistic regression analyses of risk factors for developing any stage of ROP in gestational age <33 weeks infants.

Variable	*P* value	Beta coefficient	Adjusted odds ratio	95% CI
Gestational age (weeks)	0.002*	−0.415	0.660	0.508–0.858
Birth weight (g)	<0.001*	−0.004	0.996	0.994–0.998
Maternal age (years)	0.032*	−0.104	0.901	0.819–0.991

CI: confidence interval; ROP: retinopathy of prematurity.

*P < 0.05.

**Table 3:** Univariate analyses of risk factors for the laser treatment in gestational age <33 weeks infants.

Variable	Untreated infants (*n* = 137)	Treated infants (*n* = 45)	*P* value	Odds ratio	95% CI
Gestational age (weeks)^†^	30.0 (28.3–31.8)	26.0 (24.2–27.5)	<0.001*	0.507	0.411–0.625
Birth weight (g)^ †^	1217 (901–1524)	754 (595–894)	<0.001*	0.996	0.995–0.998
Maternal age (years)^ †^	33 (30–37)	30 (28–34)	0.012*	0.917	0.852–0.986
SGA (*n*, %)	49 (36)	13 (29)	0.398	0.730	0.350–1.519
Apgar score; 1 min^†^	6 (3–8)	3 (2–5)	<0.001*	0.783	0.680–0.901
Apgar score; 5 min^†^	8 (6–9)	6 (4–7)	<0.001*	0.700	0.588–0.833
Multiple birth (*n*, %)	42 (31)	6 (13)	0.022*	0.348	0.137–0.885
Gender; Male (*n*, %)	72 (53)	23 (51)	0.866	0.944	0.481–1.852
Pulmonary surfactant (*n*, %)	57 (42)	29 (64)	0.008*	2.544	1.265–5.115

CI: confidence interval; ROP: retinopathy of prematurity; SGA: small for gestational age.

*P < 0.05.

^†^Median (interquartile range).

**Table 4:** Multivariate logistic regression analyses of risk factors for laser treatment in gestational age <33 weeks infants.

Variable	*P* value	Beta coefficient	Adjusted odds ratio	95% CI
Gestational age (weeks)	<0.001*	−0.723	0.485	0.387–0.609
Maternal age (years)	0.012*	−0.128	0.880	0.795–0.973

CI: confidence interval.

*P < 0.05.
